# Clinical Utility of MBDA Panel in the Management of Adult Onset Still’s Disease

**DOI:** 10.31138/mjr.28.3.157

**Published:** 2017-09-29

**Authors:** Xuan Gao, Olga Petryna

**Affiliations:** 1 Internal Medicine Department, Mount Sinai Beth Israel Medical Center, New York, NY, USA,; 2 NYU Langone School of Medicine, New York, NY, USA

**Keywords:** Adult Onset Still’s disease, MBDA panel, systemic, diagnosis

## Abstract

**BACKGROUND:**

Adult Onset Still’s disease (AOSD) carries an expansive presentation – typically featuring a triad of fevers, myalgia/arthralgia, and a rash – which can be mistaken for infectious or malignant etiology. As such, the role of reliable biomarkers becomes critical in the diagnosis and management of AOSD. The employment of MBDA panel may be of clinical utility in AOSD management, as we describe two cases where its application drives treatment plan.

**METHODS:**

We describe two cases where application of MBDA panel – such as Vectra DA –assisted with disease management.

**RESULTS:**

Case 1 features a 68-year-old male who presented with recurrent fevers, malaise, and a rash for three weeks. He was found to have an elevated ferritin level (15,599 ng/mL) with elevated acute phase reactants, consistent with AOSD. Vectra DA score was 77 at time of diagnosis. After treatment (see table), repeat Vectra DA at follow-up was 15. Case 2 features a 25-year-old female with history of juvenile idiopathic arthritis (then inactive) who presented with fevers, malaise, and rash. She was found to have an elevated ferritin level (321 ng/mL) with elevated acute phase reactants, also consistent with AOSD. Her Vectra DA score was 80 at peak of symptoms. She underwent appropriate treatment (see table), and repeat Vectra DA at follow-up was 17.

**CONCLUSION:**

A deeper consideration should be placed on the value of MBDA as a monitoring tool in the management of AOSD, specifically when pertaining to patient’s responsiveness to therapies.

## INTRODUCTION

Adult Onset Still’s disease (AOSD) is an auto-inflammatory disorder with an expansive and heterogeneous presentation that can often be mistaken for infectious or malignant etiology. There is no clear consensus in terms of its incidence and prevalence in different populations, but the disease is believed to have a bimodal age distribution, with its earlier peak between 15 and 25 years old, and its latter peak between 36 and 46 years old.^1^ Additionally, there are also cases of AOSD reported amongst elderly individuals exceeding 70 years old.^2^ The diagnosis is based largely on a high index of clinical suspicion, and typically hinges on a triad of symptoms that includes: recurrent, quotidian episodes of fevers of unknown origin (FUO); a characteristic maculopapular salmon-colored rash; and arthralgia/myalgia.^1–3^ These physical findings are typically corroborated by elevated acute phase reactants, such as erythrocyte sedimentation rates (ESR), C-reactive protein (CRP), and ferritin. Less common symptoms, such as sore throat, lymphadenopathy and hepato-splenomegaly, although not uncommon, remain non-specific and, oftentimes, lead to misdiagnosis. As such, the long-term management of AOSD can be difficult due to the limited amount of reliable biomarkers used to predict and manage disease activity. The introduction of multi-biomarker disease activity (MBDA) panel – such as Vectra DA – may provide a better strategy for the management of more nebulous systemic rheumatic disorders such as AOSD. The MBDA panel itself is a 12-biomarker serology study employed in the management of rheumatoid arthritis (RA).^4^ hese biomarkers include: vascular cell adhesion molecule-1 (VCAM-1), epidermal growth factor, vascular endothelial growth factor A (VEGF-A), interleukin-6 (IL-6), tumor necrosis factor receptor I (TNFRI), matrix metalloproteinase 1 (MMP-1), MMP-3, human cartilage glycoprotein 39 (YKL-40), leptin, resistin, serum amyloid, and CRP. The levels of each biomarker are incorporated into an algorithm used to generate a score that ultimately reflects the subject’s inflammatory state. This score can then be used to track disease activity and response to therapy. MBDA is not typically employed in the management of AOSD but, given that the panel’s score hinges on measurement of inflammatory markers and how AOSD features a pro-inflammatory state, there may be some clinical utility of MBDA as an assessment tool in the management of AOSD. We present two cases with the diagnosis of AOSD and the use of MBDA to track clinical progression and response to therapy.

## CASE PRESENTATION

*Patient One* was a 68-year-old male with history of hypothyroidism who presented with recurrent spiking fevers at the end of the day of unknown origin associated with generalized malaise, fatigue, lymphadenopathy and a maculopapular rash in bilateral upper extremities for three weeks. In the outpatient setting, patient was pan-CT scanned with results only remarkable for bladder thickening. He denied any familial history of rheumatological disorders, and was found to have negative ANA and rheumatoid factor (RF). There was no leukocytosis on initial inpatient lab work, and further infectious and malignant work-up was negative. Patient was found to have elevated ferritin level of 15,599 ng/mL, ESR 82 mm/hr, and CRP 18 mg/dL; and was diagnosed with Adult-Onset Still’s Disease (AOSD). Vectra DA score was 77 at time of diagnosis. He was started on pulsed steroid with resolution of symptoms, and then discharged home to complete a tapered steroid regimen with the addition of Rilonacept; repeat Vectra DA score at three-month follow-up was 15, with ESR 7mm/hr and CRP 1.4 mg/dL and resolution of presenting symptoms.

**Table 1.** Case details.

	**CASE 1**	**CASE 2**
Age, Gender	68, Male	25, Female
Presentation	Fevers, generalized malaise, rash, lymphadenopathy	Fevers, generalized malaise, arthralgia, rash, sore throat
Initial Vectra DA^*^	77 (on presentation)	80 (at peak of symptoms)
Interventions	Corticosteroids, Rilonacept	Corticosteroids, Rilonacept
Repeat Vectra DA, after therapy and at 3-months follow-up	15	17

* When applied to management of Rheumatoid Arthritis, Vectra DA score of 1–29 indicates low disease activity, 30–44 indicates moderate disease activity, and 45–100 indicates high disease activity.

*Patient Two* was a 25 year-old female with history of juvenile idiopathic arthritis (achieved remission for the past 14 years) who presented with recurrent quotidian fevers, sore throat, generalized malaise and arthralgias, and a new-onset maculopapular rash of the inner forearms and thighs bilaterally for one-month duration. Her outpatient blood work revealed WBC 20.8 k/uL but further infectious work-up was negative. She was found to have negative RF, ANA but elevated ferritin level of 321 mg/mL, ESR 75 mm/hr, CRP 138 mg/dL. Patient was diagnosed with AOSD, and started on pulsed oral steroid with methotrexate outpatient, but failed treatment. Her Vectra DA score was 80 at the peak of her symptoms. She was referred to our inpatient institution for further management, and received pulsed IV corticosteroids during her course with symptomatic improvement. Patient was discharged home on a tapered steroid regimen and Rilonacept. A repeat Vectra DA score at three-month follow-up was 17, with ESR 6mm/hr and CRP 7.4mg/dL and, also, resolution of presenting symptoms.

## DISCUSSION

Like other systemic rheumatic disease, the specific etiology of AOSD is unknown, but it is believed to have a genetic component as prior studies have indicated a relationship with HLA antigens.^1,3^ It is also suggested that alterations of cytokine production may share a role in AOSD onset.^1,2^ This speculation is fairly substantiated by the robust reduction in Vectra DA scores our two patients had while receiving Rilonacept, a long-acting inter-leukin-1 inhibitor Trap.

Nevertheless, the diagnosis of AOSD can be a challenging one as it lacks definitive diagnostic testing. Traditionally, there is marked elevation of acute phase reactants –ESR, CRP – in virtually all AOSD patients. Ferritin elevation can be seen in 70% of AOSD patients, with values typically exceeding 3000 ng/mL (normal limits 40–200 ng/mL).^1,2^ There are still a lot of cases of AOSD presenting without elevation of Ferritin levels, which may complicate initial diagnosis and make management of disease activity a struggle. Other common hematological abnormalities include: leukocytosis, anemia, and thrombocytosis,^2^ which were not observed in *Case 1*, but *Case 2* featured leukocytosis.

To date, there are at least seven sets of diagnostic criteria proposed for AOSD, but the Yamaguchi criteria (**Table 2**) carries the highest sensitivity for AOSD and is, thus, more accepted.^1^ *Case 1* fulfills three major criteria and one minor criteria; whereas *Case 2* fulfills four major criteria and 2 minor criteria. As anticipated, both cases carry a high diagnostic likelihood of AOSD; however, the long-term management and monitoring of disease progression is often dependent on continuous monitoring of inflammatory markers and patient’s self-reports of new fevers, rash, and/or arthralgia. This can be difficult as it relies on subjective recall that is confounded by a patient’s threshold for pain and suspicion of disease recurrence.

**Table 2.** Yamaguchi Diagnostic Criteria for Adult Onset Still’s Disease.

***Yamaguchi Diagnostic Criteria for Adult Onset Still’s Disease*** Requires presence of five features, with at least two major diagnostic criteria.		
	**CASE 1**	**CASE 2**
Major Criteria		
Fevers at least one week	X	X
Arthralgia lasting two weeks or longer	X	X
Rash	X	X
Leukocytosis		X
Minor Criteria		
Sore throat		X
Lymphadenopathy		
Hepatomegaly or splenomegaly		
Abnormal liver function studies		
Negative antinuclear antibody (ANA) and rheumatoid factor (RF)	X	X

As such, there is, perhaps, some utility for the use of MBDA panels in the management of AOSD, which, as mentioned, is a 12-biomarker study that is traditionally employed in RA management.^5^ If it is suggested that an elevated MBDA score represents higher RA disease activity,^6,7^ it is not unreasonable to extrapolate that an elevated MBDA score also indicates a greater inflammatory state, which is seen in active AOSD. In *Case 1*, patient’s MBDA score was 77 at time of diagnosis, which is corroborated by his elevated ESR, CRP, and ferritin. With appropriate interventions, his MBDA score decreased to 15 at his three-month follow-up date, implying improvement in disease activity and favorable response to therapy. In *Case 2*, she had previously failed Methotrexate (MTX), but there was no MBDA score obtained at time of initial diagnosis. Rather, a MBDA score performed at the peak of her symptoms – after MTX failure – yielded 80. Because of her disease progression, she required a more aggressive treatment than *Case 1*. At three-month follow-up, her MBDA score decreased to 17, also suggesting marked improvement in AOSD status and, most importantly, positive response to therapy.

In conclusion, while many strides have been made in the initial diagnosis and management of AOSD, a deeper consideration should also be placed on the value of MBDA as a disease activity tool in the monitoring of the AOSD disease course, specifically when it pertained to evaluating patient’s responsiveness to therapies. Moreover, with the advent of MBDA and other analogous panels, it has become even more important to evaluate novel and additional roles of such markers in the care and surveillance of other obscure rheumatic disease.

## INFORMED CONSENT AND DISCLOSURE NOTE

Participating patients provided written informed consent; subjects were not financially reimbursed and understood sharing of clinical vignette was strictly for educational purposes.

Additionally, authors of this publication do not hold any financial relationships with Regeneron or Amgen or any agencies promoting rilonacept or anakinra, and do not have a conflict of interest at the time of publication.
